# ADAR-Mediated A>I(G) RNA Editing in the Genotoxic Drug Response of Breast Cancer

**DOI:** 10.3390/ijms25137424

**Published:** 2024-07-06

**Authors:** Yanara A. Bernal, Eduardo Durán, Isidora Solar, Eduardo A. Sagredo, Ricardo Armisén

**Affiliations:** 1 Centro de Genética y Genómica, Instituto de Ciencias e Innovación en Medicina (ICIM), Facultad de Medicina Clínica Alemana Universidad del Desarrollo, Santiago 7610658, Chile; ybernalg@udd.cl (Y.A.B.); isolar@udd.cl (I.S.); 2 Subdepartamento de Genómica y Genética Molecular, Sección Genética Humana, Instituto de Salud Pública de Chile, Avenida Marathon 1000, Ñuñoa, Santiago 7780050, Chile; edduran47@gmail.com; 3 Department of Microbiology, Tumor and Cell Biology (MTC), Karolinska Institutet, SE-171 77 Stockholm, Sweden; sagredo1989@gmail.com; 4 Science for Life Laboratory, Department of Molecular Biosciences, The Wenner-Gren Institute, Stockholm University, SE-171 77 Stockholm, Sweden

**Keywords:** RNA editing, A>I(G), ADAR1, drug response, drug resistance, breast cancer, splicing alteration, immune response, DNA damage repair

## Abstract

Epitranscriptomics is a field that delves into post-transcriptional changes. Among these modifications, the conversion of adenosine to inosine, traduced as guanosine (A>I(G)), is one of the known RNA-editing mechanisms, catalyzed by ADARs. This type of RNA editing is the most common type of editing in mammals and contributes to biological diversity. Disruption in the A>I(G) RNA-editing balance has been linked to diseases, including several types of cancer. Drug resistance in patients with cancer represents a significant public health concern, contributing to increased mortality rates resulting from therapy non-responsiveness and disease progression, representing the greatest challenge for researchers in this field. The A>I(G) RNA editing is involved in several mechanisms over the immunotherapy and genotoxic drug response and drug resistance. This review investigates the relationship between ADAR1 and specific A>I(G) RNA-edited sites, focusing particularly on breast cancer, and the impact of these sites on DNA damage repair and the immune response over anti-cancer therapy. We address the underlying mechanisms, bioinformatics, and in vitro strategies for the identification and validation of A>I(G) RNA-edited sites. We gathered databases related to A>I(G) RNA editing and cancer and discussed the potential clinical and research implications of understanding A>I(G) RNA-editing patterns. Understanding the intricate role of ADAR1-mediated A>I(G) RNA editing in breast cancer holds significant promise for the development of personalized treatment approaches tailored to individual patients’ A>I(G) RNA-editing profiles.

## 1. Introduction

RNA editing is a post-transcriptional modification process that plays a key role in the diversification of the transcriptome, the modulation of the immune response, and the regulation of gene expression and protein function. The family of Adenosine Deaminase Acting on RNA (ADAR) enzyme catalyzes the conversion of adenosine (A) to inosine (I), modifying the mRNA interaction, and it changes the recognized nucleoside to guanosine (G) using the translation machinery (A>I(G) RNA editing). Recently, the dysregulation of A>I(G) RNA editing has been related to some human diseases, such as cancer, and their mechanisms. Regarding the drug resistance in breast cancer (BC) [1], the dynamics and implications of ADAR1-mediated A>I(G) RNA editing are not clear. This review focuses on ADAR1-mediated A>I(G) RNA editing on the genotoxic drug response in BC. For this purpose, we address the following topics: the mechanism of ADAR1-mediated A>I(G) RNA editing, its role in DNA damage repair and immune response modulation, its influence on phenotype development, and response to anti-cancer drugs in BC, including immunotherapy. Additionally, we briefly address the techniques and approaches employed in identifying A>I(G) RNA editing events and their future applications in oncology clinical practice. Revealing the functional and mechanistic impacts of ADAR1-mediated A>I(G) RNA editing in BC on the response to genotoxic drugs could reveal a new source of potential novel therapeutic strategies and molecular markers for better prognosis and treatment outcomes in patients with BC.

## 2. ADAR1-Mediated A>I(G) RNA Editing

High-throughput technologies have allowed the development of a new field known as the “epitranscriptomics” [2]. Epitranscritomics consists of biochemical RNA modifications that can affect the transfer (tRNA), ribosomal (rRNA), messenger (mRNA), small (sRNA), and long non-coding RNA (lncRNA) molecules [3]. In mammals, RNA editing is a common post-transcriptional process of great relevance due to its implications in human diseases [4]. RNA editing was first identified in 1986 in the mitochondrial DNA of kinetoplastid trypanosoma mitochondria, in which the production of RNA sequences that were not equivalent to DNA was observed, consisting of an insertion/deletion of uridine. This inconsistency was called “RNA editing” [5].

RNA editing is a modification of nucleoside sequences of the RNA transcript [6]. RNA editing involves two main deaminations, cytosine to uridine (C>U) catalyzed by APOBEC protein family (Apolipoprotein B mRNA Editing enzyme, Catalytic polypeptide-like) in DNA and RNA [7] and, on the other hand, the adenosine to inosine (A>I), where inosine is usually interpreted by the ribosome as guanosine (G), known as A>I(G); this conversion is catalyzed by a family of adenosine deaminases acting on RNA (ADARs) that act on double-stranded RNA (dsRNA). While the impact of APOBEC on human diseases and cancer has garnered significant attention due to its direct influence on DNA, research on ADAR-mediated RNA editing remains limited [7].

In mammals, A>I(G) RNA editing is the most frequent RNA modification, occurring in more than 90% of the genome [8,9]. Usually, the A>I(G) RNA-editing sites affect non-coding regions, 3’UTRs, and Alu repeat elements, which are the most abundant repetitive sequences in the human genome [10,11]. Alu repeat elements are susceptible to A>I(G) RNA editing due to similar repeat sequences that can form dsRNA. However, next-generation sequencing technologies have also allowed the identification of A>I(G) RNA editing events in non-Alu-elements [12].

ADAR family members consist of two principal domains: a N-terminal dsRNA binding domain that directly interacts with dsRNA molecules, and a C-terminal region where the deaminase domain is located [13]. ADAR1 has two isoforms. ADARp110 (110-kDa) is expressed in most tissues and is primarily located in the nucleus, where it binds to Z-DNA. However, in response to specific cellular signals, ADAR1p110 undergoes phosphorylation, which facilitates its transport from the nucleus to the cytoplasm. ADARp150 (150-kDa) has two Z-DNA binding and nuclear export signals. Additionally, ADARp150 is inducible by interferon and is co-expressed only in the presence of ADARp110 [14]. The A>I(G) RNA editing activity of ADAR1 is regulated by small ubiquitin-like modifier (SUMO)-1 [15] and is controlled by tumor interferon and ADAR copy number (1q amplification), which can explain 53% of the variability of ADAR1 expression [16]. Also, ADAR2 is expressed mostly in the brain and testis and does not contain a Z-DNA binding domain. ADAR3 has an arginine-rich domain but not a Z-DNA binding domain, and it is localized mainly in the nervous system.

ADAR1 has gained relevance among researchers due to its editing activity. The molecular consequences of ADAR1 activity depend on the region of the RNA molecule targeted. A>I(G) RNA editing events that occur in coding regions can lead to the formation of edited proteins that may present alterations in their function such as specific changes in their amino acid sequence [17] and/or also modify the secondary structure of the RNA [18]. On the other hand, when/if they occur in non-coding regions (introns or 5‘untranslated regions (UTR) and 3’UTR), these modifications can affect splicing and transcript stability [19,20]. ADAR1 can impact splicing through the A>I(G) RNA editing of cofactors and/or spliceosome components, but ADAR1 can influence splicing in an A>I(G) RNA editing-independent manner. It has been demonstrated that ADAR can influence splicing without the conversion of A>I(G). This was studied using catalytically inactive ADAR, which revealed splicing events due to the interaction of the enzyme with other splicing machinery components [21]. Both mechanisms can either create or disrupt splicing sites [22,23]. Recent studies have shed light on the potential molecular consequences of these RNA modifications with far-reaching implications, such as disruptions in key processes such as DDR and immune response. These interconnected processes, if disturbed, involve a significant impact of A>I(G) RNA editing on how cancer cells respond to drugs [24].

### 2.1. The role of ADAR1 in DNA Damage Repair

Recent reports have shown that the A>I(G) RNA editing mediated by ADAR1 plays a role in DNA damage repair (DDR) pathways [25]. This becomes relevant when considering that tumor cells are constantly exposed to sources of DNA damage, such as genotoxic drugs. This damage induces various DNA repair mechanisms, including mismatch repair (MMR) for replicative error, base-excision repair (BER) for single-strand DNA breaks (SSBs), non-homologous end-joining (NHEJ) for double-strand DNA breaks (DSBs), and homologous recombination (HR) for DSBs [26].

**Figure 1:**
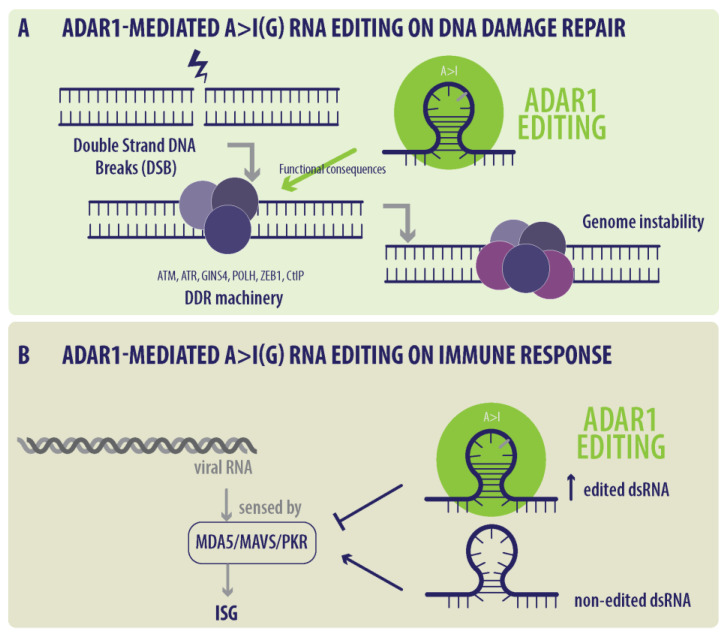
Scheme of ADAR1-mediated A>I(G) RNA editing. (**A**) ADAR1 activity function in DNA damage repair machinery such as ATM, ATR, GINS4, POLH, ZEB1, and CtIP from double-strand DNA breaks (DSB); (**B**) effect of ADAR1-mediated A>I(G) RNA-edited dsRNA and non-edited dsRNA on MDA5/MAVS/PKR function and I Interferon-Stimulated Genes (ISGs). Adapted from Nakahama T. and Kawahara Y. [30].

A>I(G) RNA editing and ADAR1 can trigger a complex DDR pathway involving genes of cell cycle modulation, apoptosis, and the replication stress response machinery, among others [26,27]. Additionally, both the A>I(G) RNA-edited site in the 3’UTR of *ATM* and another site in the intronic region of *ZEB1* are involved in the *p53* interaction network, which can affect DNA repair and the cell cycle [28]. This finding is consistent with previous results showing RNA editing in the 3’UTR of *ATM*, *GINS4,* and *POLH* in BC tumors, the DDR pathways, and cell cycle-related genes [29]. This modification potentially affects protein function in repairing DNA damage, suggesting that RNA editing might play a previously unknown role in regulating DDR pathways (Figure 1A).

Recent research introduces RNA-Editing Damage Response (REDAR) as a new layer in the DDR system. REDAR involves A>I(G) RNA editing in response to DNA breaks. This process relies on key proteins such as the checkpoint kinase ATR and the recombination factor CtIP [9]. This response is relevant for maintaining genomic stability, and ADAR2 edits DNA hybrids to facilitate their dissolution, when decreases in ADAR2 affect the genome instability and impair HR [9]. Also, this evidence suggests that DNA damage, induced by factors such as ionizing radiation or drugs, regulates the frequency of specific A>I(G) RNA-editing events [9], highlighting its significant role in the DDR.

DNA damage induces the acute translocation of ADAR1p110, preventing apoptosis [31], and recent evidence shows that ADAR1p110 activates the ATR pathway to eliminate R-loops [9]. R-loops are nucleic acid structures, formed by RNA early during DNA transcription, that hybridize with the template DNA strand, which can induce replicative stress, leading to structural collapse and inducing DSBs and genomic instability [32]. The genome stability of cancer cells is reportedly pro-oncogenic [33]. These findings show an interplay between A>I(G) RNA editing, ADAR1, and DDR. According to a recent publication, ADAR1 and ADAR2 knockdown renders cells hypersensitive to genotoxic agents, increases genomic instability, and hampers homologous recombination by impairing DNA resection [9].

### 2.2. A>I(G) RNA Editing and Immune Response

It is now widely demonstrated that A>I(G) RNA editing has a substantial impact on modulating the immune response in health and disease [30,34]. ADAR1 and other RNA-editing enzymes can modulate the expression of several immune-related genes, modulating the immune response in several types of cancer. The first study implicating the role of ADAR1-mediated A>I(G) RNA editing and the immune response came from the developmental biology field in 2009. Under normal conditions, these dsRNAs function as danger signals, triggering an innate immune response initiated and characterized by an increased expression of I Interferon-Stimulated Genes (ISGs) and promoting the response of IFN response type 1 (IFN-I), inflammation, and the recruitment of immune system cells, thereby promoting tumor destruction [34,35] (Figure 1B). In mouse models, Hartner, J.C., et al. showed that ADAR KO was lethal to embryos, and that these embryos had an increased IFN-related gene expression signature [35]. Interestingly, this phenotype can be partially rescued by *PKR* deletion [36] and rescued at birth after the deletion of MDA5 or MAVS, sensors of dsRNA [37,38].

In cancer research, one of the first reports linking A>I(G) RNA editing and immune responses was that by Zhang M. et al. in 2018 [39]. The authors elegantly showed that peptides generated by ADAR-mediated A>I(G) RNA editing can be effectively presented and recognized by tumor-infiltrating CD8+ T cells in melanoma tumors. Interestingly, they also showed that edited peptide-specific tumor-infiltrating lymphocytes (TILs) infiltrate and kill melanoma tumor cells in vivo, suggesting that ADAR1 could have dual roles as an oncogene, and indirectly, promote TIL-specific tumor cell destruction [39]. Additionally, from the A>I(G) RNA editing profiles in the TCGA database and in vitro experiments, it was evident that this difference impacts mRNA abundance and diversity, implicating ILF3, a known immune-related protein, in the regulation of this editing process [40]. For a more comprehensive review of the role of ADAR1-mediated A>I(G) RNA editing in the immune response, please see [34,41].

## 3. The Role of ADAR1-Mediated A>I(G)RNA Editing in BC

Both the expression and editing activity of ADAR have been implicated in BC. ADAR1 expression has been reported to be significantly higher in tumor tissue than in normal tissue across several cancer types [42]. Compared with those of the ADARp150 isoform, the RNA expression and abundance of the ADARp110 isoform are greater in BC cell lines [43]. Interestingly, they also showed that ADAR1 or ADAR2 KD results in Epithelial–Mesenchymal Transition (EMT), suggesting a dual role of ADAR1 and ADAR2. The authors also suggest that, although ADAR1 KD could promote EMT in vitro, ADAR expression alone may not be enough to explain A>I(G) RNA editing as a whole process.

Also, the dysregulation of ADAR1 activity has been linked to various diseases, including neurological disorders and cancer [44,45]. BC is the second most prevalent cancer, following head and neck squamous cell carcinoma, with a higher proportion of differential overediting in A>I(G) RNA-edited sites [46]. A study analyzing 81 breast cancer (BC) samples from The Cancer Genome Atlas (TCGA) revealed a significant 24% increase in RNA-editing events compared to normal breast tissue. Notably, this editing was particularly enriched within the 3’UTRs of mRNA, known targets of the ADAR1 enzyme [29]. RNA editing, a process that can significantly alter gene expression by modifying the transcriptome, can influence various aspects of tumor biology, including growth, survival, proliferation, progression, metastasis, heterogeneity, and the immune response when A-to-I(G) RNA editing is dysregulated [18,47].

Additionally, A>I(G) RNA-edited sites influences gene expression of genes related to processes such as DNA damage, immunity, DNA replication, and BC cell progression [20,48]. In patients with BC from TCGA, 9% of 7849 patients with BC had ADAR1 amplification [49,50]. Patients with ADAR1 amplification (1q amplification) have higher ADAR1 expression and an increased level of A>I(G) RNA editing compared to patients without this amplification [16]. Thus, patients with a high level of A>I(G) RNA editing exhibit poor survival [51,52,53]. High ADAR1 expression is positively correlated with the frequency of A>I(G) RNA editing and with a lower survival [16]. Therefore, A>I(G) RNA-edited sites have a cumulative impact on the cancer cell transcriptome, significantly influencing BC progression [54]. Notably, ADAR1 is specifically involved in the development of the triple-negative BC phenotype because it affects the expression of immune-related long non-coding RNAs such as LINC000944, which is associated with BC [55].

A>I(G) RNA-edited sites can affect the final gene product, and due to their location, they can have different functional consequences. With respect to A>I(G) RNA editing in coding regions with non-synonymous amino acid changes, certain A>I(G) RNA-edited sites have been linked to protein-level diversity, a phenomenon observed in BC, and some have been classified as clinically significant in cancer [56]. Some specific A>I(G) editing sites have already been strongly associated with cancer hallmarks. Preclinical studies have shown that specific A>I(G) RNA-edited sites such as NEIL1^K242R^ are widely involved in the initiation and promotion of many cancers, but this phenomenon has not been replicated in BC, neither at the in vitro nor the in vivo level [57].

Also, splicing alterations caused by ADARs could influence tumorigenesis [23,58], potentially generating isoforms that contribute to various biological mechanisms of carcinogenesis [59]. When A>I(G) RNA editing occurs in non-coding regions such as the 3′UTRs of transcripts such as ATM, GINS4, and POLH, it can affect the stability of transcripts associated with the DNA damage response (DDR) in BC cell lines [29].

ADAR1, A>I(G) RNA editing, and specific A>I(G) RNA-edited sites have gained significant relevance in understanding the biological mechanisms underlying cancer. We identified a subset of selected A>I(G) RNA-edited sites that could promote carcinogenesis processes in BC (Table 1).

## 4. The Role of ADAR1-Mediated A>I(G)RNA Editing on Anti-Cancer Drug Responses in BC

The dysregulation of ADAR1 and A>I(G) RNA-editing patterns at specific A>I(G) RNA sites are emerging as potential mechanisms of therapy resistance. Increased A>I(G) RNA-editing activity could induce functional changes that could alter the response to drugs in BC. The inhibition of ADAR1 promotes drug sensitivity to bromodomain and extra-terminal domain (BET) inhibitors by stabilizing c-Myc [64], but these studies predominantly emphasize ADAR1 expression, setting aside A>I(G) RNA-editing activity and specific A>I(G) RNA-edited sites [42]. These sites can modify proteins and not only contribute to the development of the cancer phenotype but also facilitate the evasion of drug-mediated apoptosis. This alteration in therapeutic targets could result in the elimination of drug-binding domains, ultimately inducing resistance to therapy [65]. It can even increase the stability of mRNAs, thereby activating carcinogenic pathways. For instance, an A>I(G) RNA-edited site in the 3’UTR of SCD1 has been associated with chemoresistance to 5-fluorouracil and cisplatin, and a high signature of edited SCD1 mRNA and a high ADAR1 proteomic level predict a poor prognosis in patients with gastric cancer [66]. Additionally, increased RNA-editing levels of PODXL, along with increased exon inclusion, has been associated with reduced cell migration, contributes to cisplatin chemoresistance, and is even associated with clinical outcomes in patients with Kidney Renal Clear Cell Carcinoma; however, this has not been verified in patients with BC [67]. Such mechanisms have been extensively described in the context of various genotoxic drugs utilized for cancer treatment [68]. To date, some specific A>I(G) RNA-edited sites have been described to be involved in the drug response in BC (Table 2).

Additionally, *ARSD*, *ZNF791*, *MED18*, and *RAD1* in the 3’UTR have been previously proposed to be relevant sites for predicting the overall survival (OS) and disease-free survival (DFS) in patients with BC [71], which are indirect measures of therapy response [72]. Additionally, our group described A>I(G) RNA-edited sites associated with drug sensitivity in cell lines, which were also reported in tumor samples from patients with breast cancer, where hyper-editing at a site in the *LSR* and hypo-editing in *SMPDL3B*, *HTRA4*, and *LL22NC03-80A10.6* are associated with poor progression of breast cancer, as evaluated through progression-free survival [73]. These data show the potential role of editing sites in explaining drug response, which could be involved in therapy resistance mechanisms.

### A>I(G) RNA Editing and ADAR1 in the Immunotherapy Response in BC

Immunotherapy, particularly immune checkpoint inhibitor (ICI) therapy such as anti-PD-1 or anti-PD-L1 therapy, has made significant strides in treating certain cancers, such as lung cancer and melanoma, leading to improved patient survival rates. Recently, FDA-approved anti-PD-1 immunotherapy for early-stage and metastatic triple-negative BC (TNBC) has shown promising results [74]. Recent studies have linked RNA editing and the overexpression of ADAR1 to the immunotherapy response and prognosis in various cancers [20,55,57,75,76,77,78]. ADAR1 has been identified as a potential therapeutic target for enhancing immunotherapy responses in melanoma [79], laying the foundation for studying the role of ADAR1 in immunotherapy modulation across various tumor models.

Even though, in BC, there is no direct link between ADAR1-mediated A>I(G) RNA editing and immunotherapy response, it has been described in other cancers that ADAR1 overexpression increases dsRNA editing in several cancers, decreasing their immunogenicity, and promoting tumor progression. A>I(G) RNA editing increases cellular transcriptomic and proteomic diversity, potentially enhancing neoantigen production and tumor mutational burden (TMB), a known predictor of immunotherapy response [34,39,40,52,80]. When edited by ADAR1, these dsRNAs lose their immunogenicity, the IFN-I response is inhibited, tumor infiltration decreases, and the tumor becomes less sensitive to immunotherapy with ICIs. Also, these studies demonstrated that by inhibiting the expression of ADAR1 in tumor cells, the expression and sensing of immunogenic dsRNAs (by MDA5 and PKR) is favored, reestablishing the expression of ISGs and the IFN-I response and restoring sensitivity to therapy with ICIs [37,81,82]. These suggest that ADARs could have been implicated in immunotherapy responses in certain cancer types through two primary mechanisms: neoantigen production and the IFN pathway.

**Figure 2:**
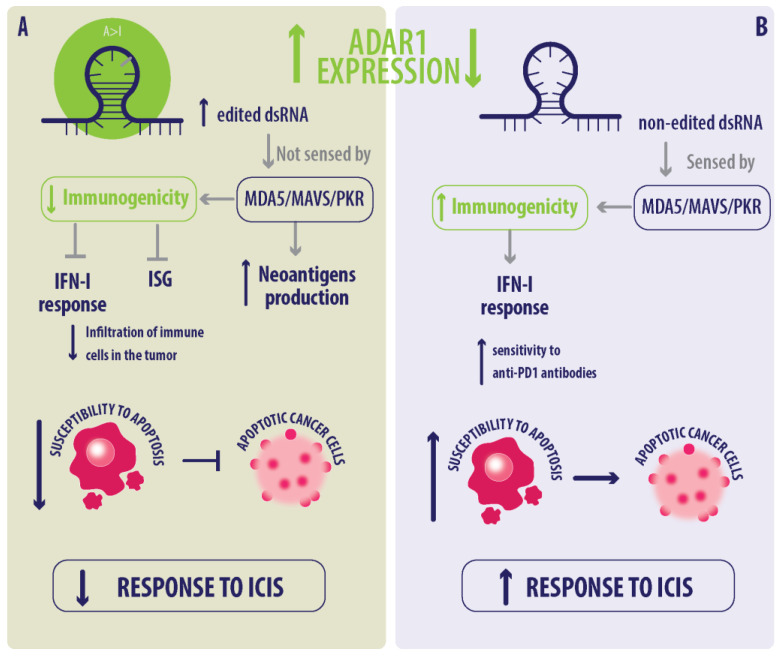
Impact of ADAR1 expression on immunotherapy response. (**A**) High ADAR1 expression reduces immunogenicity and increases neoantigen production, leading to decreased susceptibility to apoptosis and a reduced response to immune checkpoint inhibitors (ICIs). (**B**) Low ADAR1 expression enhances immunogenicity and increases susceptibility to apoptosis, resulting in an improved response to ICIs. Adapted from Nakahama T. and Kawahara Y. [30].

The role of ADAR1 in the immunotherapy response primarily involves neoantigen production, alteration in tumor cell antigen receptors, and triggering of immune responses. A characteristic of tumors is the presence of immune cells, which has been considered a central point in the selection of therapies; when there is low infiltration of immune cells, they are cold tumors, and when there is high infiltration, they are hot tumors [83]. BC is often classified as a cold tumor [83,84] with lower neoantigen loads compared to other cancers such as melanoma and lung cancer [85,86]. Komatsu and collaborators have described that the enhanced A>I(G) RNA-editing activity, induced by chemoradiation therapy, can improve the responder to ICIs by neoantigen with artificially increased production in cold induced tumors [80]. This effect is due to the promotion of proteomic diversity by RNA A>I(G) RNA editing. However, most studies support that ADAR1 inhibition and low editing activity overcome resistance to ICIs [81] (Figure 2A). In the IFN pathway, Gannon and collaborators demonstrated that, in lung cancer models, the therapeutic effect of ADAR1 deletion depends on the basal expression of ISGs and an active IFN response. This indicates that cell lines with basal sensitivity to the IFN pathway are responsive to ADAR1 inhibition, whereas those without this sensitivity are not. Interestingly, most cell lines sensitive to ADAR1 deletion correspond to the Small Cell Lung Cancer (SCLC) subtype of lung cancer. Notably, Non-Small Cell Lung Cancer (NSCLC) cell lines not sensitive to ADAR1 inhibition can be sensitized by pretreatment with IFN-α [37]. On the other hand, melanoma and other cancer models showed that deletion of ADAR1 restored sensitivity to anti-PD-1 antibodies in murine models, and this effect was dependent on the IFN machinery [37,81,82]. These studies strongly support the possibility that ADAR1 is a new therapeutic target complementary to other therapies that favors the antitumor response mediated by IFN-I, increasing or restoring the response to immunotherapy. Usually, when MDA5/MAVS/PKR senses dsRNA, it triggers an antiviral response by increasing IFN production and activating ISGs, leading to an anti-viral and anti-tumoral response. However, when dsRNA is edited by ADAR1, it cannot be sensed, disrupting the IFN and ISG production pathways, altering the immune response, and reducing susceptibility to apoptosis [37,81,82]. ADAR1 deletion in lung cancer models shows sensitivity to the basal expression of ISGs and an active IFN response, with SCLC being particularly more responsive, and the presence of ADAR1 appears to be associated with resistance to ICI therapy in certain cancers [87] (Figure 2B).

Despite these findings, further evaluation is needed to understand the link between ADAR1, A>I(G) RNA editing, and immunotherapy responses, particularly in TNBC. Genomic databases and studies focused on immunotherapy in patients with BC will be instrumental in clarifying the role of ADAR1 in enhancing or restoring immunotherapy responses.

## 5. A>I(G) RNA Editing Identification

The increasing interest in ADAR1 activity has encouraged the development of different strategies such as bioinformatics tools, quantification of the A>I(G) RNA-editing levels, and in vitro discovery or validation. However, the identification of A>I(G) RNA-edited sites through RNA sequencing and the validation of these sites remain challenging.

### 5.1. Techniques for the Identification of Site-Specific A>I(G) RNA Editing

The identification of A>I(G) RNA-editing sites began due to the development of DNA-sequencing technologies (Table 3). The emergence of next-generation sequencing (NGS) has enabled the identification of more than 2 million A>I(G) RNA-editing sites in humans [88]. The first A>I(G) RNA-editing sites were discovered using Sanger sequencing [89] to identify differences between nucleotides of DNA and cDNA in sequences of ion channel receptors in the human brain. Sanger sequencing can be very accurate, but also very tedious if used to screen entire human genes for differences between RNA and DNA [88].

In the early 2000s, with the success of the human genome project [90], the first study was conducted to search for A>I(G) RNA-edited sites using comparative genomics, examining the mRNA of highly conserved genetic sequences within 18 Drosophila species, and identifying 16 new editing sites [91]. In parallel with the use of comparative genomics for the detection of A>I(G) RNA-edited sites, databases and sequence repositories were developed that include a large public collection of cDNA transcript sequences from libraries of expressed sequence tags (ESTs) [88]. In 2004, four studies were conducted that used a systematic evaluation of A to G mismatches, the signal for A>I(G) RNA editing, aligning millions of human ESTs or full-length cDNA transcripts to genomic sequences, and identifying more than 10,000 RNA-editing sites [88].

On the other hand, in 1997, a biochemical method was developed to detect inosines in mRNAs. This method involves the specific cleavage of inosines from RNA using RNase T1 after glyoxal treatment [92]. Subsequently, in 2010, a chemical method to identify inosines, known as Inosine Chemical Erasing (ICE) sequencing, was described based on the cyanoethylation reaction of inosines, where cyanoethyl groups are added to inosines that react with hydroxylamine to produce stable residues of inosine and reverse transcription-PCR [93]. However, this method reported many false positives due to mapping errors [94]. Almost 10 years later, an article was published on the optimization of the identification of editing sites in sequencing using *Escherichia coli* Endonuclease V (eEndoV), which is capable of isolating and enriching edited transcripts in RNA before sequencing, optimizing the coverage and detectability of editing sites [95]. Later, human endonuclease V (hEndoV) was used by blocking 3′ deoxyadenosine, which produces new 3′OH terminals that can be identified by sequencing [96]. Recently, Wei and collaborators developed specific Ligation of Inosine Cleaved Sequencing (Slic-seq), a method based on RNase T1, hEndoV, and EndoVIPER-seq that has been proposed for the identification of inosines [95,97]. These innovative techniques promise to improve the detection and breadth of A>I(G) RNA-edited sites and enrich RNA-containing inosines.

In the last decade, the emergence of NGS technology has achieved the sequencing of millions of DNA fragments in a single experiment [98], allowing the identification, with high throughput, of A>I(G) RNA-editing sites. This technology has generated a 10-fold expansion of A>I(G) RNA-edited sites compared to those achieved with published ESTs [88]. In 2011, the first study that sought to identify de novo A>I(G) RNA-edited sites was carried out, and more than 10,000 exonic RNA–DNA differences (RDDs) of all 12 possible mismatch types were detected [99]. After several subsequent studies, it was shown that most of the RDDs identified by Li M and his team in the 2011 study were false positives resulting from technical artifacts such as errors introduced during reverse transcription and inaccurate sequencing read alignments [89,99,100]. The main inference from these studies was that the accurate de novo identification of A>I(G) RNA-edited sites using NGS requires meticulous methods to separate authentic A>I(G) RNA-edited sites from false ones [88]. Following this observation, in 2012 and 2013, computational processes were developed for the de novo identification of A>I(G) RNA-editing sites from coincident DNA and RNA sequencing [88]. Both the genome masking technique used to identify hyper-edited sequences and the ICE method have been successfully combined with high-throughput RNA sequencing, and the development of powerful computational processes has made the study of A>I(G) RNA editing using RNA-seq datasets a routine task [88].

In 2014, Zhang R. and his team published a new method for identifying A>I(G) RNA-edited sites, as they reported the existence of a technical limitation of RNA-seq that leads to inaccurate quantification of the allelic proportions of genes with low to moderate expression levels [101]. Faced with this situation they developed a targeted RNA sequencing method (mmPCR-seq), that combines microfluidics-based multiplex PCR and NGS that detects A>I(G) RNA editing in the same way as other sequencing methods, but due to the pre-amplification step accurately measures A>I(G) RNA-editing levels in samples with low expression levels [101]. This method complements RNA-seq and provides a highly desirable solution for future applications.

Shortly after that, to implement a rapid and cost-effective method to detect a specific A>I(G) RNA-editing fingerprint, an A>I(G) RNA editing site-specific primer design strategy compatible with qRT-PCR SYBR green protocols (RESSq-PCR) was developed [102]. The concept behind this innovative method is that it predicts that RNA-edited transcripts differ from wild-type sequences at a single nucleotide position, so the detection of edits by qRT-PCR requires highly sensitive primer design strategies [102].

In assays based on the analysis of a heterogeneous set of cells, molecules in rare or different cells may escape detection within that universe of cells. Because of this problem, Mats Nilsson and his team developed an alternative to methods based on PCR and in situ hybridization, managing to detect transcripts in situ by converting the mRNA into localized cDNA molecules that are detected with padlock probes and RCA primed in the objective [103]. They designed a panel of barcoded padlock probes to examine the levels of A>I recoding editing in a set of transcripts using multiplexed in situ chemical sequencing by observing how the editing of 14 RNA sites changes spatiotemporally during mouse brain development, revealing a previously unknown level of complexity in the regulation of RNA editing from A>I [104].

Finally, with the development of third-generation sequencing (TGS) technologies, useful tools for studying RNA biology emerged, such as Pacific Biosciences, (Menlo Park, CA, USA, PacBio), Oxford Nanopore Technologies (ONT), and long-read GIREMI (L-GIREMI) [105]. ONT is a specialized method to capture unique signatures of inosines in the direct RNA sequencing of long reads [106]. On the other hand, L-GIREMI identifies RNA-editing sites in long-read RNA sequencing data by handling sequencing errors and biases through a model-based approach [105]. These new methods aim to optimize the ability to discover edited sites with greater precision.

**Table 3:** Experimental method of A>I(G) RNA-edited sites.

“De Novo” A>I(G)-RNA-Edited Sites		
Method	Observations	Ref.
Sanger sequencing	Accurate, but very tedious to compare DNA and RNA.	[89]
Comparative genomics	-	[91]
Biochemical method (Inosine)	-	[92]
Inosine Chemical Erasing (ICE) sequencing	Many false positives.	[93,94]
eEndoV and hEndoV	To isolate and enrich edited transcripts in RNA before sequencing, optimizing the coverage of editing sites with low expression levels.	[95,96]
Slic-seq	RNase T1 + hEndoV + EndoVIPER-seq.	[97]
NGS and RNA-seq	Identification with high throughput. Requires meticulous methods to separate authentic from false results.	[98,99,100]
mmPCR-seq	Measures RNA-editing levels in samples with low expression levels. Complements RNA-seq.	[101]
Third-generation sequencing (L-GIREMI, PacBio, and ONT)	Identifies RNA-editing sites in long-read RNA-seq data.	[105,107]
Specific A>I(G)-RNA-edited sites		
Method	Observations	Ref.
RESSq-PCR	Rapid and cost-effective method and compatible with qRT-PCR SYBR. Requires highly sensitive primer design strategies.	[102]
Padlock Probe + RCA	PCR and in situ hybridization. Requires the design of a panel of barcoded padlock probes to examine specifics editing RNA sites changes. Allows spatiotemporal resolution on cells or tissues.	[103,104]

### 5.2. Bioinformatic Tools for A>I(G) RNA-Edited Site Identification

Several strategies have been developed for identifying A>I(G) RNA-edited sites using RNA-seq. The matching of DNA-seq and RNA-seq data is the optimal approach for the identification of RNA-edited sites. However, this is not always possible, so bioinformatic tools have been developed to identify sites solely from RNA-seq. An exhaustive list of tools for identifying A>I(G) RNA-edited sites is detailed in Wang 2021 [108]. The standardization of bioinformatic protocols has made it possible to accurately identify A>I(G) RNA editing. The global A>I(G) RNA editing activity through the Alu editing index (AEI), focuses on Alu sequences where most A>I(G) editing occurs in RNA [109]. This index consists of the ratio of the discordant reads on the editing site to the total reads on the site (concordant (AA (positive strand) or TT (negative strand)) plus discordant (AG (positive strand) or TC (negative strand)) in repetitive Alu elements. This approach condenses all A>I(G) RNA-editing patterns into a single index, which, if examined separately, could reveal different biological mechanisms affected by the A>I(G) RNA-edited sites.

Notably, various efforts by researchers have allowed for the compilation of high-confidence A>I(G) RNA-edited sites, which are housed in different databases, such as DARNED (database of RNA editing in flies, mice, and humans) https://darned.ucc.ie/, (accessed on 3 May 2024) [110], RADAR (rigorously annotated database of A>I(G) RNA editing) [111], MiREDiBase (miRNA Editing Database) focused on A>I(G) RNA editing of miRNA https://ncrnaome.osumc.edu/miredibase/, (accessed on 1 January 2024) [112], and LNCediting related to lnc-RNA, http://bioinfo.life.hust.edu.cn/LNCediting/, (accessed on 3 May 2024) [113]. Even interactive databases focused on cancer can query specific sites or even perform statistical calculations between normal and tumor tissues according to the level of A>I(G) RNA editing (Table 4).

In 2023, an ADAR1 signature in the Catalogue of Somatic Mutation in Cancer (COSMIC) was defined using the tri-nucleotide context of Single Nucleotide Variant (SNV) on an RNA molecule, in which the RNA Single Base Substitution (RNA SBS) 1 was defined as ADAR1 activity [119,120,121]. This approach could prove valuable for assessing ADAR1 activity across the transcriptomic profile.

Finally, another difficulty arises in the discovery of editing sites, i.e., determining whether there are differences in the editing level between two conditions such as gender, cases/controls, responders/non-responders, low/high sensitivity to a drug, or tumoral/non-tumoral tissue; it is related to a particular parameter such as TMB, age, etc. The REDIT test offers a better balance between sensitivity and false positive rates than Fisher’s exact test, the *t*-test, the thresholded *t*-test, the thresholded Wilcoxon-test, or the Wilcoxon-test. This is a robust test for identified differential A>I(G) RNA-edited sites, based on beta-binomial distribution in case–control studies comparing the edited and non-edited sites or regression analysis with continuous variables [122]. Selecting the best test would allow for an improvement in the statistical analysis when determining editing sites enriched under certain conditions.

## 6. Translational Application of ADAR1 Editing in Oncology

The interest of researchers in translating knowledge into the clinical realm and its application to oncology patients is primarily focused on countering the effects of ADAR1. There has been a search for various components for the direct inhibition of ADAR1 or even strategies such as drugs targeting editing sites of interest. 8-Azaadenosine and 8-Chloroadenosine are non-selective inhibitors of ADAR1 [123], whereas 8-Azanebularine-Modified RNA Duplexes [124] are selective inhibitors. Non-selective inhibitors block mTOR and activate AMPK in renal carcinoma [125]. This activation induces autophagy in BC cell lines through ATP depletion [126], and it improve the antileukemic activity of BCL-2 inhibition through venetoclax, a selective inhibitor of the BCL-2 protein, that targets AML [127]. Regarding selective inhibitors, a recent study demonstrated that Rebecsinib (17S-FD-895) selectively inhibits the ADAR1p150 isoform of ADAR1 [128] and inhibits stem cell propagation as a selective splicing modulator in AML [129].

However, one of the main limitations is the risk of potential off-target effects that could lead to undesired adverse effects in healthy tissues. This is critical, since ADAR1 has important functions, such as modulating innate immune function in primary macrophages [130], potentially increasing susceptibility to viral infections. Additionally, reduced ADAR1 expression has been associated with the development of severe inflammatory diseases [131,132], and may dysregulate immune responses, leading to autoimmune diseases [36], and disrupting cellular homeostasis [133]. Non-selective inhibitors of ADAR1, being adenosine analogues, can be incorporated into adjacent RNA and DNA sequences [134]. They can also inhibit DNA synthesis [135] and even integrate into the cellular ATP pool [136] and induce the activation of AMPK triggering the unfolded protein response and inducing apoptosis in other tissues [137].

Another significant challenge in ADAR1 inhibition lies in the complexity of ADAR1-mediated RNA-editing mechanisms in different cellular contexts and cancer types. In different cellular environments, the expression levels and function of ADAR1 can be affected by various factors, including the presence of inflammatory signals, hypoxic conditions, and interactions with other RNA-binding proteins [138]. Some cancer that shows higher ADAR1 expression are lung, colon, glioma, and breast cancer; conversely, lower ADAR1 has been described in melanoma and pancreatic cancer [139]. This variability highlights the necessity of comprehensively understanding the unique molecular characteristics of each tumor to effectively tailor ADAR1-targeted therapies.

Notably, directly targeting these specific sites for inhibition, rather than ADAR1 itself, may offer a more precise and effective treatment approach. Due to the discovery of A>I(G) RNA-edited sites and their correlation with clinical characteristics, it presents a novel avenue for potential therapeutic intervention. Unlike DNA base editing, analogous changes introduced in RNA are not permanent or heritable; instead, they allow reversible and doseable effects that are appealing for various therapeutic applications [140]. For instance, antisense oligonucleotides (ASOs) targeting the A>I(G) RNA-editing site complementary sequence (ECS) associated with AZIN1 effectively inhibited A>I(G) RNA editing at the site, resulting in decreased cell viability in cell lines, and reduced tumor growth in xenograft models [141]. However, currently, these strategies are not available for clinical application.

The sequencing technologies widely used in research and clinical settings focus on modifications at the DNA level, attributed to the functional alterations that cause different phenotypes in different cancer types such as gastric and lung cancer, melanoma, and acute myeloid leukemia. Nowadays, several signatures based on A>I(G) RNA-edited sites are a promising model for predicting different clinical outcomes in patients with cancer. Unregulated A>I(G) RNA editing is associated with a poor prognosis [142], and a signature of A>I(G) RNA-edited sites was useful for predicting survival and response of chemotherapy in patients with gastric cancer [143], the response to immunotherapy in patients with lung cancer [144], and survival probability in patients with acute myeloid leukemia [145]. In melanoma, a high load of A>I(G) RNA editing-derived (RED) neoantigens correlated with increased overall survival compared to a low load of RED. Additionally, the RED load was significantly higher in patients who responded to immunotherapy [146]. Research shows clinical utility in various cancers; however, they have not been specifically developed for BC. This finding demonstrates the clinical potential in oncology of ADAR1 activity signatures as clinical relevance in patients with BC [147].

## 7. Conclusions

In this review, we address the relationships among A>I(G) RNA-editing sites, ADAR1 activity, and genotoxic and/or immunotherapy responses, with a special focus on BC. RNA editing, particularly A>I(G) mediated by ADAR1, is a significant post-transcriptional modification that impacts RNA molecules, with crucial roles in human diseases. A>I(G) RNA editing in coding and non-coding regions affects gene products, influencing splicing, transcript stability, and processes like DNA damage response, which are crucial in the carcinogenesis. Furthermore, ADAR1-mediated A>I(G) RNA editing influences DNA damage repair pathways, affecting how tumor cells respond to genotoxic stress and highlighting a potential regulatory role in DDR. Moreover, ADAR1-mediated A>I(G) RNA editing modulates immune responses by altering dsRNA immunogenicity, impacting cancer cell sensitivity to immunotherapies, and influencing immune-related gene expression.

**Figure 3:**
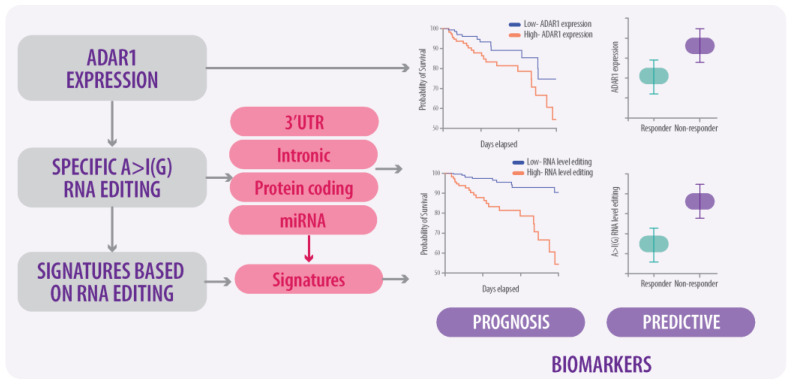
Flowchart illustrating the ADAR1-mediated RNA-editing application from the molecular consequences to clinical applications as prognosis and predictive biomarkers in BC.

In BC, we address the dysregulation of ADAR1, which is overexpressed in tumor tissues and affects various aspects of tumor biology including growth, survival, and metastasis. Specifically, ADAR1 expression correlates with poor survival, particularly influencing the triple-negative BC phenotype. Additionally, we explore the role of ADAR1-mediated A>I(G) RNA editing on genotoxic and immunotherapy responses. ADAR1 overexpression reduces dsRNA editing, leading to decreased immunogenicity and promoting tumor progression. Dysregulation of ADAR1 and A>I(G) RNA editing at specific sites can induce therapy resistance in breast cancer (BC) by altering drug response mechanisms. Thus, increased RNA editing activity can lead to functional changes that affect drug-binding domains, mRNA stability, and the evasion of drug-induced apoptosis. While a direct link between ADAR1-mediated RNA editing and immunotherapy response in BC is not established, ADAR1’s role in enhancing neoantigen production and the IFN pathway suggests potential therapeutic targeting to improve immunotherapy outcomes (Figure 3).

Additionally, we discussed bioinformatic strategies including the use of algorithms and analysis tools to predict and detect A>I(G) RNA-editing sites in large genomic and transcriptomic datasets. These tools help identify patterns and evaluate the frequency and location of editing events. Moreover, experimental strategies encompass laboratory techniques such as RNA sequencing and RNA editing assays to validate and characterize A>I(G) RNA-editing sites identified bioinformatically. These techniques are essential for confirming the presence and functionality of editing sites in specific biological contexts. Finally, we discuss the potential clinical implications of A>I(G) RNA editing; researchers are exploring ADAR1 inhibitors and targeting specific RNA-editing sites to improve cancer treatments, though challenges like off-target effects and tumor variability must be addressed, with current applications showing promise primarily in cancers other than BC.

## Figures and Tables

**Table 1:** A>I(G) RNA-edited sites involved in the carcinogenic processes of BC.

A>I(G) RNA-Edited Site/Gene	Consequence	Phenotype of BC	Type of Study	Reference
Chr8:103841636/AZIN1	S367G	Promote cell proliferation	Cell lines	[16]
Non-edited GABRA3	I342M	Promote breast cancer cell migration, invasion and metastasis	Cell lines, animal models	[60,61]
Chr1:160302244/COPA	I164V	Promote cell viability, migration, and invasion	Cell lines	[56]
Chr3:58156064/FLNB	M2293V	Tune down the growth- and invasion-suppressing activities of FLNB	Cell lines	[54]
miR-140-3p target on DFFA	miRNA	Apoptosis	Cell lines	[62]
hsa-miR-200b target on ZEB1/2 and LIFR	miRNA	Worse patient survival cell invasion and migration by ZEB1/2 and LIFR inhibition. Negative correlation with TP53	Patients with BC Cell lines	[63]

**Table 2:** A>I(G) RNA-edited sites involved in the drug response in BC.

RNA-Edited Sites/Gene	Region	Drug Response	Type of Study	Reference
miR25-3p miR125a-3p/DHFR	miRNA	Resistance to methotrexate	In vitro	[69]
Chr19:58355670/ZNF587B	Coding	Resistant to doxorubicin	Cell line data	[70]
Chr20:36147563/BLCAP	Coding	Resistant to doxorubicin	Cell line data	[70]
Chr8:103841636/AZIN1	Coding S367G	Low sensitivity to IGF-1 R BMS536924	Patients with BC, in vitro	[56]
Chr4:158281294/GRIA2	Coding R764G	High sensitivity to MEK Cl1040	Patients with BC, in vitro	[56]
Chr13:46090371/COG3	Coding I635V	High sensitivity to MEK Trametinib	Patients with BC, in vitro	[56]

**Table 4:** Databases of A>I(G) RNA-edited sites in cancer.

Source	Link	Reference
CAeditome	https://ccsm.uth.edu/CAeditome/, (accessed on 3 May 2024)	[114]
Editome Disease Knowledgebase	https://ngdc.cncb.ac.cn/edk/, (accessed on 3 May 2024)	[115]
The Cancer Editome Atlas	http://tcea.tmu.edu.tw, (accessed on 3 May 2024)	[116]
RNA Editing Interactive Analysis (REIA)	http://bioinfo-sysu.com/reia/, (accessed on 3 May 2024)	[117]
Genetic and pharmacogenomic landscape of RNA editing in cancers (GPEdit)	https://hanlaboratory.com/GPEdit/, (accessed on 3 May 2024)	[118]
